# Identification of New Regions in HIV-1 gp120 Variable 2 and 3 Loops that Bind to α4β7 Integrin Receptor

**DOI:** 10.1371/journal.pone.0143895

**Published:** 2015-12-01

**Authors:** Kristina K. Peachman, Nicos Karasavvas, Agnes-Laurence Chenine, Robert McLinden, Supachai Rerks-Ngarm, Kaewkungwal Jaranit, Sorachai Nitayaphan, Punnee Pitisuttithum, Sodsai Tovanabutra, Susan Zolla-Pazner, Nelson L. Michael, Jerome H. Kim, Carl R. Alving, Mangala Rao

**Affiliations:** 1 U.S. Military HIV Research Program, Walter Reed Army Institute of Research, Silver Spring, MD, United States of America; 2 Henry M. Jackson Foundation for the Advancement of Military Medicine, Bethesda, MD, United States of America; 3 United States Army Medical Component, Armed Forces Research Institute of Medical Sciences, Bangkok, Thailand; 4 Ministry of Public Health, Bangkok, Thailand; 5 Data Management Unit, Mahidol University, Bangkok, Thailand; 6 Royal Thai Army, Armed Forces Research Institute of Medical Sciences, Bangkok, Thailand; 7 Vaccine Trials Center, Mahidol University, Bangkok, Thailand; 8 Veterans Administration New York Harbor Health Care System and NYU School of Medicine, New York, United States of America; Emory University School of Medicine, UNITED STATES

## Abstract

**Background:**

The gut mucosal homing integrin receptor α4β7 present on activated CD4^+^ T cells interacts with the HIV-1 gp120 second variable loop (V2). Case control analysis of the RV144 phase III vaccine trial demonstrated that plasma IgG binding antibodies specific to scaffolded proteins expressing the first and second variable regions (V1V2) of HIV envelope protein gp120 containing the α4β7 binding motif correlated inversely with risk of infection. Subsequently antibodies to the V3 region were also shown to correlate with protection. The integrin receptor α4β7 was shown to interact with the LDI/V motif on V2 loop but recent studies suggest that additional regions of V2 loop could interact with the α4β7. Thus, there may be several regions on the V2 and possibly V3 loops that may be involved in this binding. Using a cell line, that constitutively expressed α4β7 receptors but lacked CD4, we examined the contribution of V2 and V3 loops and the ability of V2 peptide-, V2 integrin-, V3-specific monoclonal antibodies (mAbs), and purified IgG from RV144 vaccinees to block the V2/V3-α4β7 interaction.

**Results:**

We demonstrate that α4β7 on RPMI8866 cells bound specifically to its natural ligand mucosal addressin cell adhesion molecule-1 (MAdCAM-1) as well as to cyclic-V2 and cyclic-V3 peptides. This binding was inhibited by anti-α4β7-specific monoclonal antibody (mAb) ACT-1, mAbs specific to either V2 or V3 loops, and by purified primary virions or infectious molecular clones expressing envelopes from acute or chronic subtypes A, C, and CRF01_AE viruses. Plasma from HIV-1 infected Thai individuals as well as purified IgG from uninfected RV144 vaccinees inhibited (0–50%) the binding of V2 and V3 peptides to α4β7.

**Conclusion:**

Our results indicate that in addition to the tripeptide LDI/V motif, other regions of the V2 and V3 loops of gp120 were involved in binding to α4β7 receptors and this interaction was blocked by anti-V2 peptide, anti-V2 integrin, and anti-V3 antibodies. The ability of purified IgG from some of the uninfected RV144 vaccinees to inhibit α4β7 raises the hypothesis that anti-V2 and anti-V3 antibodies may play a role in blocking the gp120-α4β7 interaction after vaccination and thus prevent HIV-1 acquisition.

## Background

The HIV field has expended great efforts to determine the mechanism(s) of protection observed in the HIV-1 RV144 phase III clinical trial. Immune correlate analysis and subsequent secondary analysis showed that antibodies against the HIV-1 Env gp120 V1V2 region correlated inversely with the risk of infection [1], thus generating the hypothesis that these antibodies may have contributed to the protection. The antibodies targeted multiple binding epitopes in the V3 and V2 region including the mid-region of the V2 loop that contained conserved epitopes with the amino acid sequence KQKVHALFYKLDIVPI (HXB2 numbering sequence 169–184) [2–7]. This region included the integrin-binding motif LDI/V (residues 179–181) [8, 9].

Integrins are cell surface receptors that are involved in a number of functions including migration of cells to different tissues and cell adhesion [10, 11]. The α4β7 integrin receptor is a heterodimer consisting of an α4 subunit that can associate with either a β1 or a β7 subunit [12, 13]. Activated α_4_β_7_ integrin on lymphocytes binds to its cognate ligand MAdCAM-1 expressed on endothelial cells leading to the adhesion, extravasation, and homing of these lymphocytes to the gut tissues. The importance of the gut homing receptor α_4_β_7_ was further highlighted by the findings that it can also serve as a receptor for HIV-1 and SIV transmission leading to the up-regulation of LFA-1 and spread of HIV-1 from cell-to-cell through virological synapses [14, 15]. The α_4_β_7_ integrin receptor on activated CD4^+^ T cells provides a link between the earliest site of HIV-1 transmission, the mucosa, and the gut inductive sites where T cells are depleted during infection [16, 17].

We have recently shown in humanized DRAG mice ([Rag1KO.IL2RγcKO.NOD ("NRG") strain] with chimeric transgenes encoding for HLA-DR*0401 [HLA-DRA/HLA-DRB1*0401] fused to the I-Ed MHC- II molecule) [18] that after a vaginal HIV-1 challenge, the gut α_4_β_7_^+^CD4^+^ T cells are infected with HIV-1 with a subsequent decrease in these cells during the course of HIV-1 infection [19]. It has been previously reported that in SIV infected rhesus macaques, the depletion of β7-expressing CD4^+^T cells in the blood and in the intestine are comparable. Furthermore, a decrease in plasma and gastrointestinal viral loads was observed after administration of an α_4_β_7_ monoclonal antibody prior to and during SIV infection [20]. A strong correlation between the frequencies of memory CD4 T cells expressing high levels of α4β7 integrin and susceptibility to SIV rectal transmission has also been demonstrated [21]. The above findings therefore suggest that *in vivo*, α4β7 integrin may be playing an important role in SIV and HIV-1 transmission [22].

The interaction of HIV-1 envelope protein and the activated form of α_4_β_7_ has been thought to be through a conserved tripeptide motif LDI(V) present at the tip of V2 loop of HIV-1 gp120 [8, 23]. It has been hypothesized that the extended form of α4β7 may facilitate this interaction, which might be instrumental for the “permanent” establishment of a HIV-1 positive state [8, 9, 23]. However, unlike CD4 and CCR5, which are required for viral entry, interaction with the α_4_β_7_ receptor may not be essential for entry.

The mechanism of protection induced by anti-V2 antibodies and their influence on HIV-1 acquisition in the RV144 trial is still unclear. However, protection does not appear to be due to the neutralizing function of the antibodies [24]. Subsequently, two separate nonhuman primate studies demonstrated that antibodies directed against the V2 region reduced the acquisition of infection [25, 26]. It is possible that anti-V2 antibodies may function by either directly blocking or sterically hindering the α_4_β_7_-gp120 interaction. However, the potential implications of gp120-V2 binding to α_4_β_7_ integrin receptor in HIV transmission are still a topic of debate.

We examined the interactions between α4β7 integrin and either primary HIV-1 virions, or infectious molecular clones containing either acute or chronic envelopes, or peptides derived from the V2 and V3 regions of gp120 using RMPI8866 cells constitutively expressing the α4β7 integrin receptor. We also assessed the ability of V2- and V3-specific monoclonal antibodies (mAb) to block V2/V3 peptide-α4β7 integrin interaction. Finally, we tested the plasma of HIV-1 uninfected and infected individuals as well as IgG purified from RV144 vaccinees for the presence of α4β7 integrin blocking antibodies. Our data provide insights into new regions of HIV-1 gp120 that interact with α4β7 integrin molecule, which could have implications for future HIV-1 vaccine design.

## Methods

### Ethics statement, protocol authorization, and regulatory approval

RV144 (WRAIR Protocol #900): This clinical trial [27] protocol and all related documents were approved by the following independent Institutional Review Boards (IRBs): Division of Human Subject Protection, Walter Reed Army Institute of Research; Ethical Review Committee for Research in Human Subjects, Ministry of Public Health, Thailand. (http://clinicaltrials.gov/ct2/show/NCT00223080?term=RV144&rank=2;NCT00223080).

(WRAIR Protocol #1617): This protocol and all related documents were approved by the following independent Institutional Review Boards (IRBs): Division of Human Subject Protection, Walter Reed Army Institute of Research; Ethical Review Committee for Research in Human Subjects, Ministry of Public Health, Thailand. All volunteers provided written informed consent following discussion and counseling by the clinical study team prior to enrollment and before any trial related procedures were performed.

### Cell line and reagents

The human B lymphoma cell line RPMI8866 that constitutively expresses α4β7 on its cell surface was purchased from Sigma-Aldrich. Cells were grown at a density of 3-9x10^5^ cells/ml at 37°C and 5% CO_2_ in the following media: RPMI 1640, 10% heat inactivated fetal bovine serum, 1% penicillin/streptomycin, and 1% glutamine (purchased from Quality Biologics Inc.; except for FBS which was purchased from Gemini Bio-Products). Peptides were synthesized with or without biotin at the amino terminus of the peptide by JPT Peptide Technologies (See Table 1 for sequences and lengths). Some of the peptides were cyclized by disulfide bond formation and the purity was determined to be greater than 90% by high pressure liquid chromatography and mass spectrometry. Streptavidin and AlamarBlue^®^ were purchased from Invitrogen. MAdCAM-1 was purchased from R & D Systems. NeutrAvidin was purchased from Life Technologies Corp. The following reagents were obtained through the AIDS Research and Reference Reagent Program, Division of AIDS, NIAID, NIH: α4β7 monoclonal antibody, ACT-1, (cat#11718) from Dr. A. A. Ansari [20]. HIV-1_IIIB_ rgp120 (CHO expressed), ACT-1, a mouse monoclonal antibody that specifically binds to the α4β7 heterodimer including the active form, and pSG3Δenv were obtained through the AIDS Research and Reference Reagent Program, Division of AIDS, NIAID, NIH from Drs. John C. Kappes and Xiaoyun Wu. pSG3Δenv contains a four-nucleotide insertion mutation (CTAG) in envelope, leading to a translation stop codon after amino acid residue 142 [28, 29].

**Table 1:** Amino Acid Sequences for V2 and V3 Peptides.

*Cyclic or Linear peptide based on 92TH023 Strain*	*HXB2 amino acid numbering*	*Amino Acid Sequence (Biotin-Ttds-peptide-NH*_*2*_ *) (Bold indicates changes from the original sequence)*
Cyclic V2 (42aa)	157–196	CSFNMTTELRDKKQKVHALFYKLDIVPIEDNTSSSEYRLINC
Cyclic V2 SMR	157–196	CSFNMTTELRDK**QVLFKDIHKIVKPLYA**EDNTSSSEYRLINC
Cyclic V2 SFL	157–196	**CENLTDKMFTSR**KQKVHALFYKLDIVPI**SESRLDETNYNISC**
Cyclic V2 SCR (42aa)	157–196	**CQLYSLFIRLTKVKITELMKYSNPVHSDKIREFNTDSNDAEC**
Linear V2 (42aa)	157–196	^*^XSFNMTTELRDKKQKVHALFYKLDIVPIEDNTSSSEYRLINX
Cyclic V3 (36aa)	295–331	CTRPSNNTRTSINIGPGQVFYRTGDIIGDIRKAYC
*Cyclic peptide based on MN Strain*	*HXB2 amino acid numbering*	*Amino Acid Sequence*
Cyclic V2 (40aa)	157–179	CSFNITTSIGDKMQKEYALLYKLDIEPIDNDSTSYRLISC
Cyclic V3 (35aa)	295–330	CTRPNYNKRKRIHIGPGRAFYTTKNIKGTIRQAHC
*Cyclic peptide based on Acute C Strain C06980v0c22*	*HXB2 amino acid numbering*	*Amino Acid Sequence*
Cyclic V2 (40aa)	157–179	CSFNITTELRDKRKKEHALFNNLDIVQLDGNSSLYRLINC
Cyclic V3 (35aa)	295–330	CTRPNNNTRKSIRIGPGQTFYATGDIIGDIRQAYC

*X = S-methyl Cysteine

### Expression of α4β7 on RPMI8866 cells

RPMI8866 cells (0.5 x10^6^) were incubated for 30 minutes on ice with 10% normal goat sera in PBS to block the Fc receptors. Cells were suspended in cold FACS buffer (PBS-containing 0.5% BSA) and incubated at room temperature for 30 minutes with the mAb ACT-1 conjugated to APC (1 μg). Cells were washed twice with cold FACS buffer, fixed with 2% formaldehyde in PBS, and analyzed on a LSRII flow cytometer. The data was analyzed using FlowJo8.8.6 software (TreeStar Inc.).

### Binding of gp120 and cyclic V2 peptide to α4β7 on RPMI8866 cells

RPMI8866 cells (1 x10^6^) were suspended in cold FACS buffer (PBS-containing 0.5% BSA) and incubated at room temperature for 30 minutes with LIVE ⁄ DEAD^®^ Fixable Dead Cell Stain (Life Technologies) as per the manufacturer’s instruction. Cells were washed twice with cold FACS buffer before being incubated with 10% goat sera in PBS for 30 minutes on ice. All subsequent incubations were carried out for 30 minutes on ice. CHO- expressed HIV-1_IIIB_ rgp120 (2.5 μg) and biotinylated cyclic V2-TH023 peptide (1 μg and 5 μg) were incubated with RPMI8866 cells. Following washing twice with cold FACS buffer, polyclonal mouse anti-gp145 (sera obtained from a mouse immunized three times with gp145 clade B protein encapsulated in liposomes containing monophosphoryl lipid A; unpublished data) was then added to cells previously incubated with gp120. Cells were washed and goat anti-mouse Texas Red (Thermo Fisher Scientific-Pierce) was added. Cyclic V2 TH023 (1 μg and 5 μg) fully scrambled V2 TH023 (SCR; 1 μg and 5 μg) treated samples were incubated with neutrAvidin-PE. Cells were washed twice with cold FACS buffer, fixed with 2% formaldehyde in PBS, and analyzed on a LSRII flow cytometer. The data was analyzed using FlowJo8.8.6 software (TreeStar Inc.). Peptide sequences are shown in Table 1.

### RPMI8866 cell line dose curve

Serial two-fold dilutions of RMPI8866 cells in the log phase of growth starting at 4 x 10^5^ cells were incubated in a 96-well plate with RMPI 1640 media (1% FBS, 1% pen/strep, 1% glutamine) and AlamarBlue^®^ dye (10μL per well). The plate was incubated at 37°C (5% CO_2_) for 8 hours. Fluorescence (Excitation 560 nm and Emission 590 nm) was measured at 1 hour intervals using a M2 plate reader (Molecular Devices). The relative fluorescence units were plotted against time in hours. Data points are the mean of 6 replicates.

### Inhibition of binding of α4β7 on RPMI8866 cells to MAdCAM-1, V2 peptides, and V3 peptides

Triplicate wells of a 96-well U-bottom polystyrene plate (Immunlon) were coated overnight at 4°C with 100 μl of 2 μg/ml of MAdCAM-1 or Streptavidin diluted in 0.05 M bicarbonate buffer, pH 9.6. The streptavidin-coated plates were then incubated with biotinylated cyclic or linear V2 peptides or cyclic V3 peptides (5 μg/ml in bicarbonate buffer, See Table 1 for sequence data) for 1 hour at 37°C. The solution from the plates was discarded and the plates were then blocked with blocking buffer (25 mM TRIS, 2.7 mM potassium chloride, 150 mM sodium chloride, 0.5% casein, 4 mM manganese chloride, pH 7.2) for 1 hour at 37°C. The solution was discarded and the plates were incubated with sample buffer (25 mM TRIS, 2.7 mM potassium chloride, 150 mM sodium chloride, 4 mM manganese chloride, pH 7.2 containing 1% fetal bovine sera) for 45 minutes at 37°C. Plates were manually washed 4 times with blocking buffer followed by the addition of 2 x 10^5^ RPMI8866 cells/well that had been pre-incubated for 45 minutes at 37°C with sample buffer, with sample buffer containing 0.5 μg/ml of mAb ACT-1 or as a control, normal mouse serum (1:100). Plates were then incubated at 37°C (5% CO_2_) for 1 hour, washed 5 times with wash buffer followed by the addition of 100 μl of RPMI-1640 containing 1% FBS, pen/strep/glutamine. The adhered cells were detected by the addition of 10 μl of AlamarBlue^®^ dye. Plates were incubated at 37°C (5% CO_2_) for 8 hours. Fluorescence was measured at 2 hour intervals using a M2 plate reader. For each condition, a minimum of 2 to 12 independent experiments run in duplicate or triplicate was performed. The optimum amount of coating proteins/peptides and the amount of ACT-1 required to inhibit α4β7 on RPMI8866 cells was determined in separate experiments (data not shown). Peptide sequences are shown in Table 1.

### Competitive inhibition of binding of α4β7 on RPMI8866 cells to MAdCAM-1 by cyclic V2 and V3 peptides, primary HIV-1, and Infectious Molecular Clones (IMCs)

96-well plates were coated with MAdCAM-1 as described above. RPMI8866 cells (2 X 10^6^/ ml) were incubated with cyclic V2 or V3 MN, TH023, or acute subtype C (C-6980v0c22) peptides or with scrambled flanking region (SFL) or the mid region (SMR) TH023 V2 or with a completely scrambled (SCR) TH023 peptide (10 μg/ml) for 45 minutes at 37°C before being added in triplicate to the MAdCAM-1 coated plates for 1 hour at 37°C. In concurrent experiments, RMI8866 cells were incubated with purified primary HIV-1 (0.75 ng to 6 ng/well p24) subtype B (SF162, US-1, JRFL), subtype CRF01_AE viruses (92TH023), IMCs (0.75 ng to 1000 ng/well p24 in each case) containing acute or chronic envelopes (Acute CRF01_AE (703357) [GenBank:JN944658], C (C-6980v0c22) [GenBank:HM215344], A(851891); Chronic CRF01_AE (CM235.2) [AF259954.1], C (ET02220LucR) [U46016]) [30–32] or with pSG3Δenv. pSG3Δenv contained a four-nucleotide insertion mutation (CTAG) in Env, leading to a translation stop codon after amino acid residue 142. The pSG3Δenv clone is routinely used for generating Env pseudotyped infectious virions.

Viruses were tested in triplicate in two independent experiments. In additional experiments, mannose, HIV-1 p24, and ovalbumin (0.2–10 μg/ml) were used in the competition assay as nonspecific protein controls (data in Table 2; 3–7 experiments performed in triplicate), whereas, an anti-RSV mAb, Synagis^®^ was used as a nonspecific antibody control. Synagis^®^ was incubated for 45 minutes with either MAdCAM-1 or with RPMI8866 cells and then placed in the assay system. The remainder of the assay was similar to that described above. Significance was determined by paired Student’s t test.

**Table 2:** Specificity of the Binding Inhibition.

Concentration	Mannose	p24	Ovalbumin
μg/ml	% Inhibition ± SD	% Inhibition ± SD	% Inhibition ± SD
0.2	6.7 ± 7.6	8.2 ± 5.6	7.2 ± 12.5
1.0	7.6 ± 8.8	14 ± 9.9	2.0 ± 3.5
5	16.3 ± 14.9	9.1 ± 8.7	13.6 ± 11.8
10*	0	0	18

No significant inhibition of α4β7 integrin binding to MAdCAM-1 in a competition assay by mannose, p24, and ovalbumin was observed. The data represent the average of 3–7 experiments performed in triplicate.

*The data represent the average of triplicate determinations from a single experiment for this concentration.

### Inhibition of binding of α4β7 on RPMI8866 cells to MAdCAM-1 by V2 or V3 specific mAbs, plasma from HIV-1 uninfected and infected individuals, or purified IgG from vaccinated individuals

Streptavidin-coated plates were incubated with biotinylated cyclic V2 TH023 peptide (5 μg/ml in bicarbonate buffer as described above) for 1 hour at 37°C. RPMI8866 cells (2 X 10^6^/ml) were incubated with V2-specific mAbs (CH58, CH59), V2i mAbs (830A, 2158), V3 specific mAbs (2219, 2557) or with a non-V2-specific mAb CH54 (5–50μg/ml), plasma from HIV-1 uninfected or naturally infected individuals (WRAIR protocol #1617), or with purified IgG (WRAIR Protocol #900) (10 μg/ml) from RV144 pre and post-vaccinated (6 month) individuals for 45 minutes at 37°C before being added in triplicate to the peptide coated plates for 1 hour at 37°C. The rest of assay is similar to that described in detail above. CH58, CH59, and CH54 are human monoclonal antibodies isolated from RV144 vaccinees [33]. 830A, 2158, 2219, and mAb 2557 are also human mAbs. The mean of triplicates is shown in the graphs.

## Results

### Expression of α4β7 on RPMI8866 cells and binding to HIV-gp120 V2 peptide

**Fig 1:**
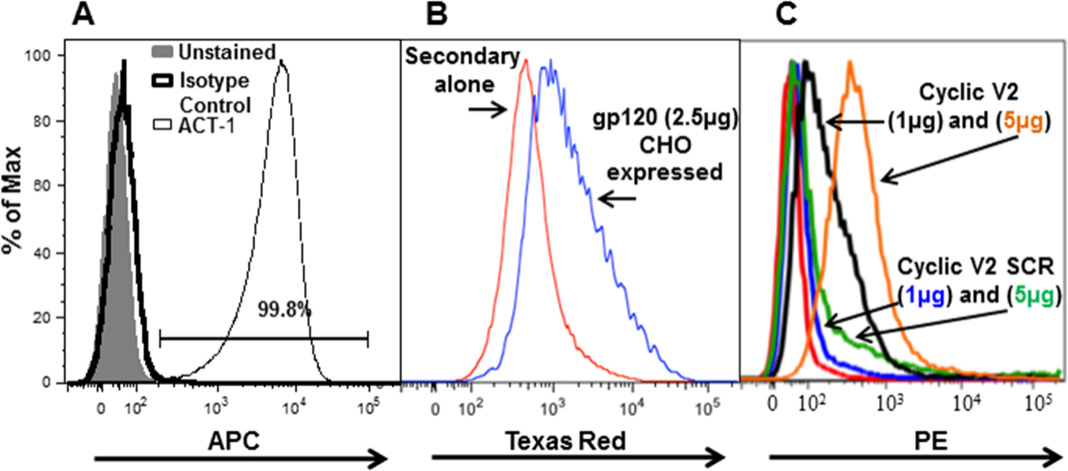
Binding of mAb ACT-1, HIV-1 gp120, and cyclic V2 peptides to RMPI8866 cells. **(A)** RPMI8866 cells (0.5 x10^6^) were incubated with 1 μg APC labeled ACT-1 (α4β7 specific antibody). Antibody binding was measured by flow cytometry and is presented as a histogram. Over 99% of the RPMI8866 cells expressed α4β7 integrin. Unstained cells (grey shading) and isotype control antibody (solid line) were used as controls. **(A and C)** Histograms show the binding of CHO-expressed HIV-1 gp120 (2.5 μg, blue line panel **B),** biotin labeled cyclic V2 TH023 peptide (1μg and 5 μg; black and orange lines, respectively; panel **C**), and biotin labeled cyclic V2 TH023 -fully scrambled peptide (SCR; 1 μg and 5 μg; blue line and green line; panel **C**) to RMPI8866 cells. Polyclonal mouse anti-gp145 antibody followed by goat anti-mouse-Texas Red was used to visualize the gp120 protein binding while the V2 peptides were visualized with neutrAvidin-PE. Cells treated with only anti-mouse-Texas Red (red line, panel **B**) and unstained cells (red line, panel **C**) were used as controls in addition to the fully scrambled V2 peptide (blue and green line, panel **C**). Representative histograms are shown for panels **A**, **B**, and **C** (n = 2).

The human B-cell lymphoma cell line, RPMI8866, has been reported to stably express integrin α4β7 on its cell surface [34]. In order to determine the percentage of RPMI8866 cells that constitutively express α4β7, cells were incubated with APC-labeled ACT-1 antibody and then analyzed on a LSRII flow cytometer (Fig 1A; S1 Data, S2 Data and S3 Data). ACT-1 is a conformational-specific anti-α4β7 mouse mAb, which binds the α4β7 heterodimer including the active form. Over 99% of RPMI8866 cells bound ACT-1, demonstrating that these cells constitutively express the functional form of α4β7. Each cell expressed approximately 100,000 α4β7 receptors on its cell surface (data not shown). It has been demonstrated that the V2 loop of HIV-1 envelope protein gp120 binds to the activated form of α4β7 on CD4+ T lymphocytes [8]. To determine if α4β7 integrin on RMPI8866 cells could bind to gp120 envelope protein and also to the V2 loop, cells were incubated with CHO-expressed subtype B gp120 (Fig 1B; S4 Data and S5 Data), biotinylated cyclic V2 92TH023 peptide (subtype CRF01_AE; henceforth referred to as TH023, see Table 1 for sequence), or biotinylated cyclic fully scrambled (SCR) V2 TH023 peptide (Fig 1C; S6 Data, S7 Data, S8 Data, S9 Data and S10 Data) followed by the addition of polyclonal mouse anti-gp145 sera and Texas red-labeled goat anti-mouse antibody (Fig 1B; S5 Data) or neutrAvidin-PE (Fig 1C; S6 Data, S7 Data, S9 Data and S10 Data) and then analyzed by flow cytometry. Both gp120 protein (Fig 1B) and cyclic V2 TH023 peptide (Fig 1C) bound to α4β7 expressed on the surface of RPMI8866 cells, while the SCR peptide (S6 Data and S9 Data) showed similar binding as the unstained cells (Fig 1C; S8 Data).

### Inhibition of binding of α4β7 to MAdCAM-1 by mAb ACT-1

**Fig 2:**
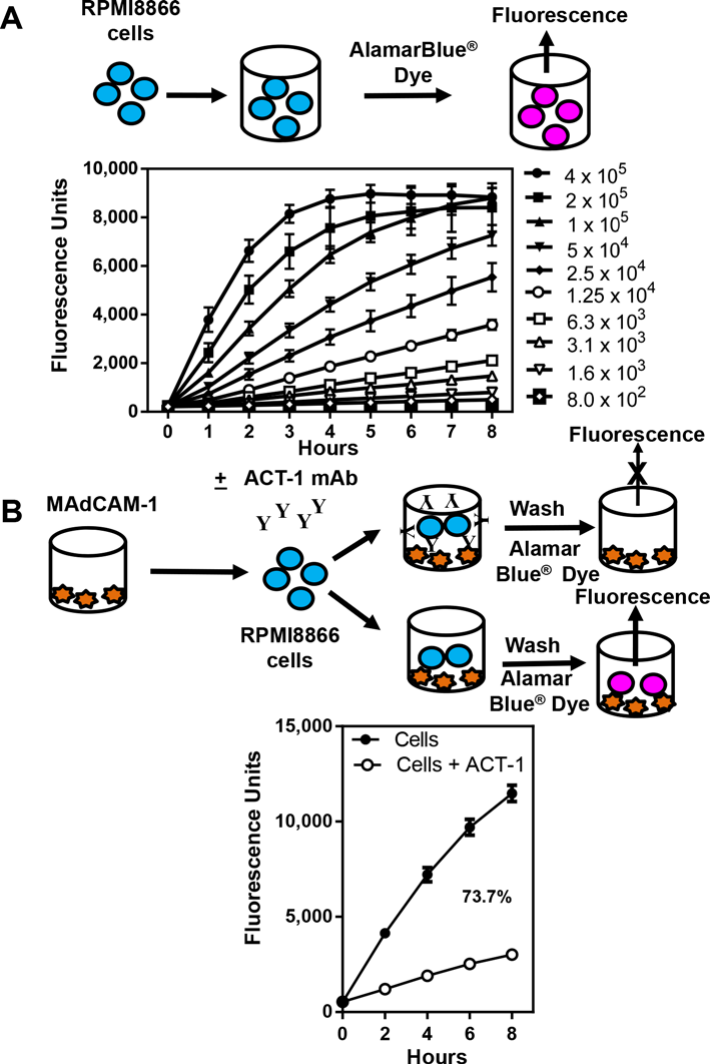
RPMI8866 cell titration curves and inhibition of binding of α4β7 integrin receptor to MAdCAM-1. (**A**) The top of the Fig shows a schematic representation of the experiment. Varying numbers of RPMI8866 cells in media were added to 96-well U bottom plates followed by the addition of AlamarBlue^®^ dye. Cell numbers ranged from 800–400,000 cells per well. The plate was incubated at 37°C in a CO_2_ incubator. Fluorescence was measured for 8 hours at 1 hour intervals using a M2 plate reader (excitation 560 nm; emission 590 nm). The data are plotted as relative fluorescence units as a function of time. The average ± S.D. of six replicates for each time point are shown. (**B**) Schematic of the assay set up. RMPI8866 cells were incubated with media only (solid circles) or with ACT-1 (0.5 μg/well; open circles, left panel) and then added to MAdCAM-1 (natural ligand of α4β7 integrin; 0.2 μg/well) coated plates. The plates were washed and 100 μl of media and 10 μl of AlamarBlue^®^ dye were added to each well. Fluorescence was measured immediately after the addition of the dye (time 0) and then at 2 hours intervals for 8 hours. The percent inhibition at the 8 hour time point is shown. The data are plotted as relative fluorescence units as a function of time and represent the average ± SEM of 12 experiments done in triplicate.

In order to utilize this cell line to detect interactions between proteins, peptides, viruses, and antibodies to α4β7, we standardized an *in vitro* assay system for quantitating bound cells using AlamarBlue^®^, a water-soluble, nontoxic, and permeable reagent that contains resazurin, an oxidation-reduction indicator, which is reduced by metabolically active cells to resorufin, a highly fluorescent red compound. The magnitude of fluorescence signal quantified by a fluorometer is proportional to the number of live bound cells. Varying numbers of RMPI8866 cells in 2-fold dilutions starting at 400,000 cells/ well were plated in RMPI-1640 -phenol free media containing 1% FBS, pen/strep, and glutamine. AlamarBlue^®^ dye was added and fluorescence was measured at 1 hour intervals for 8 hours. As shown in Fig 2A, the fluorescence was dose-dependent. As the cell number increased, there was a proportional increase in the level of fluorescence due to increased reduction of resazurin. Depending on the metabolic state of the cell, the fluorescence can range from 9,000 to 12,000 units at the 8 hour time point. Fluorescence units below 1,000 was considered as background fluorescence. For the three highest numbers of cells (100,000, 200,000, and 400,000), the fluorescence reached a plateau before the 8 hour time point. We chose 200,000 cells/well for all further experiments unless mentioned otherwise.

In order to determine if MAdCAM-1, the natural ligand of α4β7 integrin, could bind to α4β7 on the surface of RPMI8866 cells, 96-well plates were coated with MAdCAM-1. After blocking the wells, RPMI8866 cells pre-incubated in the absence (closed circles) or presence of mAb ACT-1 (open circles) were added to the wells, washed, and the binding of α4β7 on RPMI8866 cells to MAdCAM-1 was detected by the fluorescence of the added AlamarBlue^®^ dye (Fig 2B, schematic diagram and graphs). Pre-incubation of RPMI8866 cells with ACT-1 mAb would block the interaction of α4β7 with MAdCAM-1, thus reducing the number of RPMI8866 cells that would adhere to the plate. This would result in a reduced fluorescence signal. The data are plotted as fluorescence units over time and represent the average of 6–12 experiments done in duplicate or triplicate ± SEM. In the absence of mAb ACT-1 (closed circles), approximately 12,000 fluorescence units were obtained by 8 hours representing 100% binding, while a decrease in the fluorescence units in the presence of mAb ACT-1 indicated an inhibition of binding of α4β7 integrin to its ligand. Based on this, the percent inhibition was calculated. ACT-1 inhibited the binding of α4β7 to MAdCAM-1 by 74%, while the control (normal mouse sera at a dilution of 1:100) did not inhibit the binding, thus showing the specificity of inhibition (data not shown).

### Inhibition of binding of α4β7 to MAdCAM-1 by HIV-1

**Fig 3:**
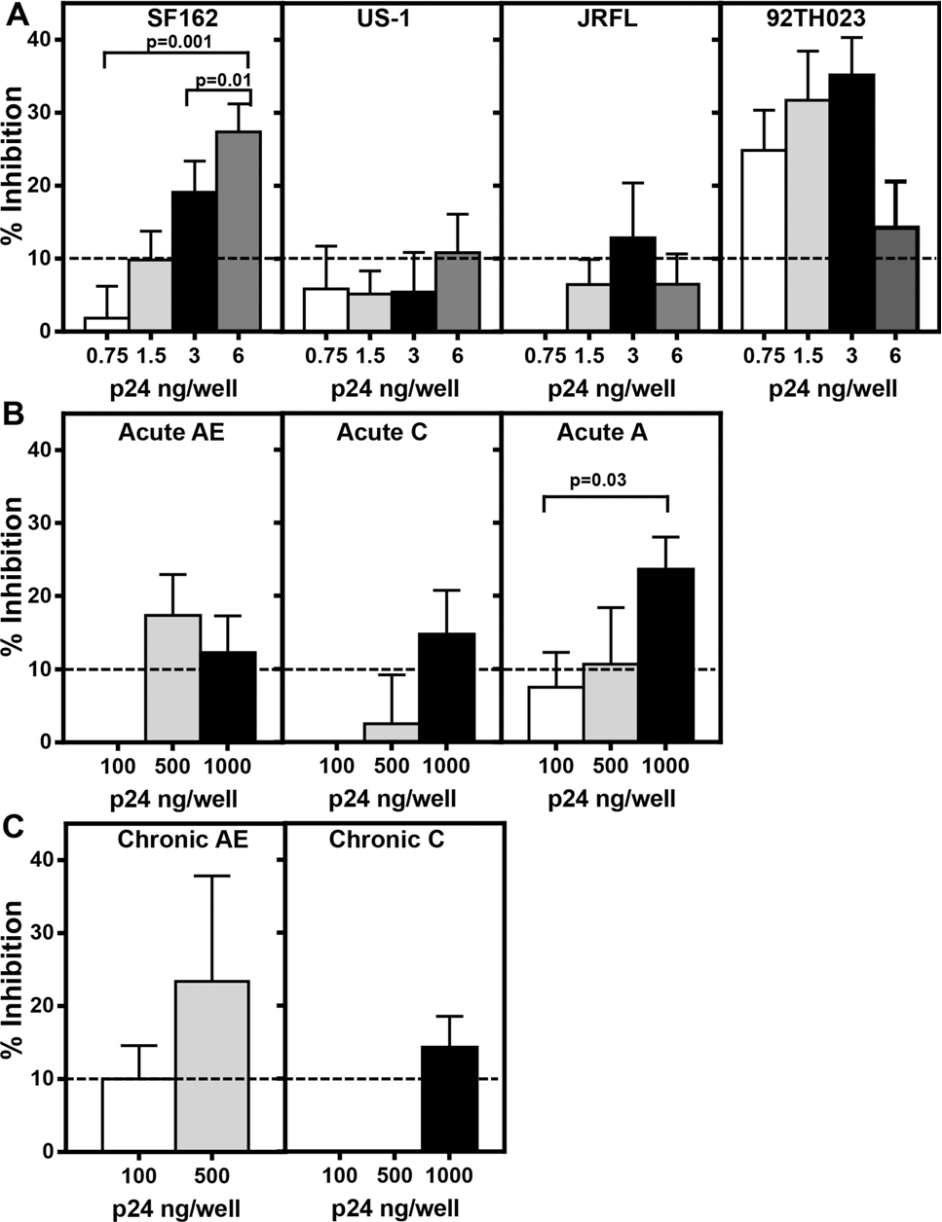
Inhibition of α4β7 integrin binding by primary virus and IMC’s containing acute or chronic envelopes. Increasing concentrations of **(A)** purified primary subtype B viruses, SF162 (left panel), US-1 (second panel), JRFL (third panel) and subtype CRF01_AE (92TH023; right panel), **(B)** IMCs with acute envelopes (subtype CRF01_AE left panel, subtype C center panel, and subtype A right panel), or **(C)** IMCs with chronic envelopes (subtype CRF01_AE left panel and subtype C right panel) were pre-incubated with RPMI8866 cells before being added to MAdCAM-1 coated plates. Primary viruses, except US-1 and JRFL, and IMCs inhibited the binding of α4β7 on RMPI8866 cells to MAdCAM-1 at one or more concentrations of the virus tested. The average percent inhibition of α4β7 integrin binding ± SEM as a function of p24 concentration is plotted for two independent experiments assayed in duplicate or triplicate for each virus or IMC. The dotted line represents the cut off value as determined by using a pSG3Δenv IMC with a frame shift in the envelope region. Significance was determined by paired Student’s t test.

In order to demonstrate that HIV-1 envelope protein on the surface of virions is capable of binding α4β7 and thus inhibiting the binding of α4β7 to MAdCAM-1, varying amounts (based on p24 concentrations) of purified primary viruses including subtype B (SF162, US-1, JRFL), subtype CRF01_AE (92TH023; Fig 3A), full-length infectious molecular clones (IMCs) from acute viruses from subtypes CRF01_AE, C, and A (Fig 3B), and chronic IMC viruses from subtypes CRF01_AE and C (Fig 3C) were pre-incubated with RPMI8866 cells and then added to MAdCAM-1-coated plates (Fig 3). As a control, pSG3Δenv IMC with a frame shift in the envelope region was used in the assay at concentrations of p24 ranging from 0 to 500ng. The average inhibition of binding of α4β7 to MAdCAM-1 by pSG3Δenv IMC virus was 10 ± 2%. This cut off value is represented as a dotted line in all of the panels of Fig 3. As shown in Fig 3A (panel 1), a significant dose-dependent inhibition of binding of α4β7 on RPMI8866 cells to MAdCAM-1 was observed in the presence of SF162 virions (p = 0.001 and p = 0.01 comparing 6 ng/well vs 0.75 ng/well and 3 ng/well, respectively), demonstrating that SF162 virions bind to α4β7 on RPMI8866 cells and block its interaction with MAdCAM-1. A dose-dependent increase in inhibition of binding was also observed with 92TH023 (Fig 3A, right panel) with inhibition (approximately 25–35%) observed at a p24 concentration of 0.75–3 ng /well. In contrast, a dose-dependent inhibition of α4β7 binding by US-1 and JRFL was not observed (Fig 3A, second and third panels). The acute and chronic IMCs showed varying degrees of inhibition at the concentrations of the virus tested, whereas the chronic subtype C IMC virus inhibited the binding only at the highest concentration of the virus tested. Compared to the primary viruses, a much higher concentration of IMCs was required to inhibit α4β7 binding to MAdCAM-1. These results show that the envelope protein present on the surface of the virions is capable of binding α4β7 on RPMI8866 cells and thus prevent the binding to MAdCAM-1 to varying degrees.

### Binding of α4β7 integrin to HIV-1 envelope gp120 V2 and V3 peptides

**Fig 4:**
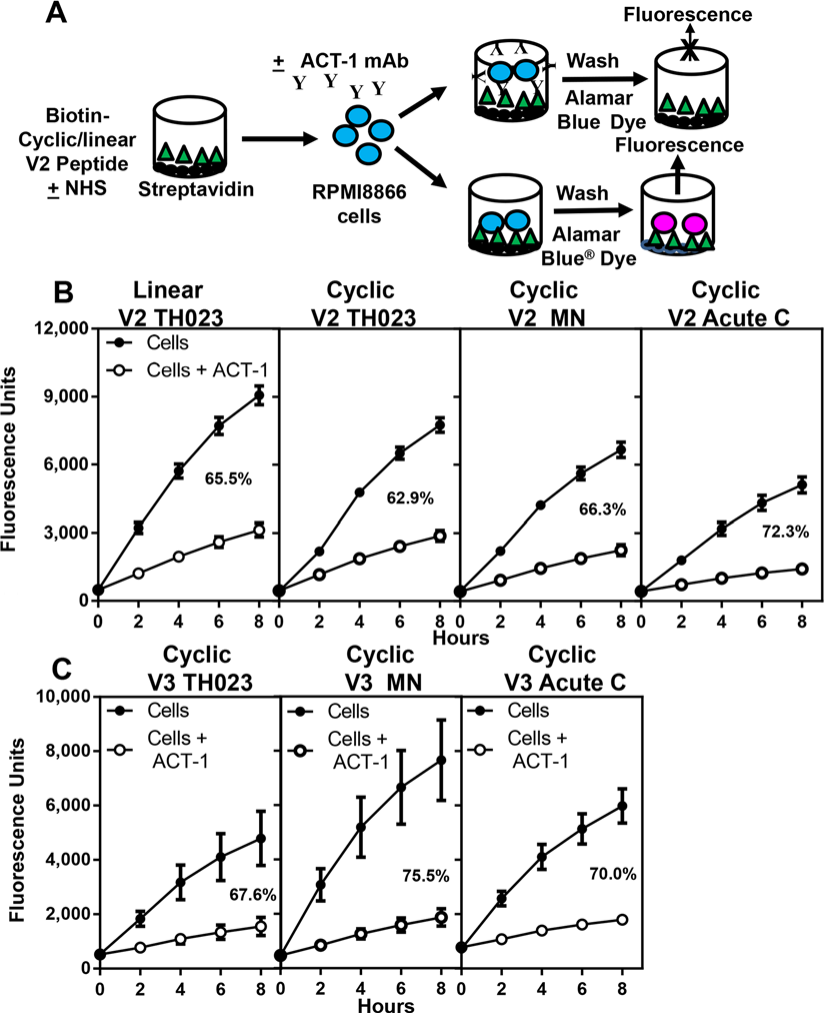
Binding of α4β7 integrin receptor to cyclic and linear V2 peptides. **(A)** Schematic of the assay set up. **(B and C)** RMPI8866 cells were incubated with media (solid circles), ACT-1 (open circles) and then added to (**B**) linear (left panel), cyclic V2 peptides (TH023, MN, and acute C, second, third and right panels, respectively), or (**C**) cyclic V3 peptides (TH023, left panel, MN, middle panel, and acute C, right panel) coated plates respectively. The plates were washed and 100 μl of media and 10 μl of AlamarBlue^®^ dye was added to each well. Fluorescence was measured immediately after the addition of the dye (time 0) and then at 2 hour intervals for 8 hours. The percent inhibition at the 8 hour time point is shown in each panel. The data are plotted as relative fluorescence units as a function of time and represent the average ± SEM of 3 to12 experiments done in duplicate or triplicate.

It has been previously reported that the binding of α4β7 integrin receptor to HIV-1 envelope protein is through the V2 region [8]. Using our assay, we examined whether cyclic and linear V2 peptides could bind to α4β7 on RMPI8866 cells and whether the binding could be inhibited by ACT-1. The sequences of the gp120 V2 peptides (TH023, MN, and Acute C (C06980v0c22)) used are presented in Table 1. A schematic of the assay design is shown in Fig 4A. Plates, 96-well U-bottom, were coated with streptavidin followed by biotinylated linear or cyclic V2 peptides. After blocking the wells, RPMI8866 cells pre-incubated in the absence (closed circles) or presence of mAb ACT-1 (open circles) were added to the wells, washed, and the binding of α4β7 on RPMI8866 cells to the V2 peptides was measured by the fluorescence of resorufin (Fig 4B). Depending on the ability of α4β7 on RPMI8866 cells to bind the various peptides tested, the fluorescence varied from 3,000 to 9,000 units. The data are plotted as fluorescence units over time and represent the average of 6–12 experiments done in duplicate or triplicate ± SEM. The percent inhibition was calculated as described above and ranged from 63–72% for the various peptides tested (Fig 4B). Similar degrees of inhibition were obtained with both linear and cyclic TH023 peptides. The control normal mouse sera in 4 independent experiments done in triplicate showed an inhibition of 2 ±1% (data not shown).

Similar experiments were performed with biotinylated cyclic V3 peptides derived from subtypes CRF01_AE (TH023), B (MN), and acute subtype C (C06980v0c22) viruses. V3 peptides bound α4β7 integrin on RPMI8866 cells (Fig 4C), which could be specifically inhibited by ACT-1 (68–76% inhibition). The above data demonstrate that synthetic linear and cyclic V2 and cyclic V3 peptides derived from subtypes CRF01_AE, B, and acute C bind to α4β7 integrin on RPMI8866 cells and can be readily measured using our assay system. The binding is specific and can be inhibited by ACT-1.

### Competition assay using V2 and V3 peptides and characterization of the V2 peptide region that interacts with α4β7 integrin

**Fig 5:**
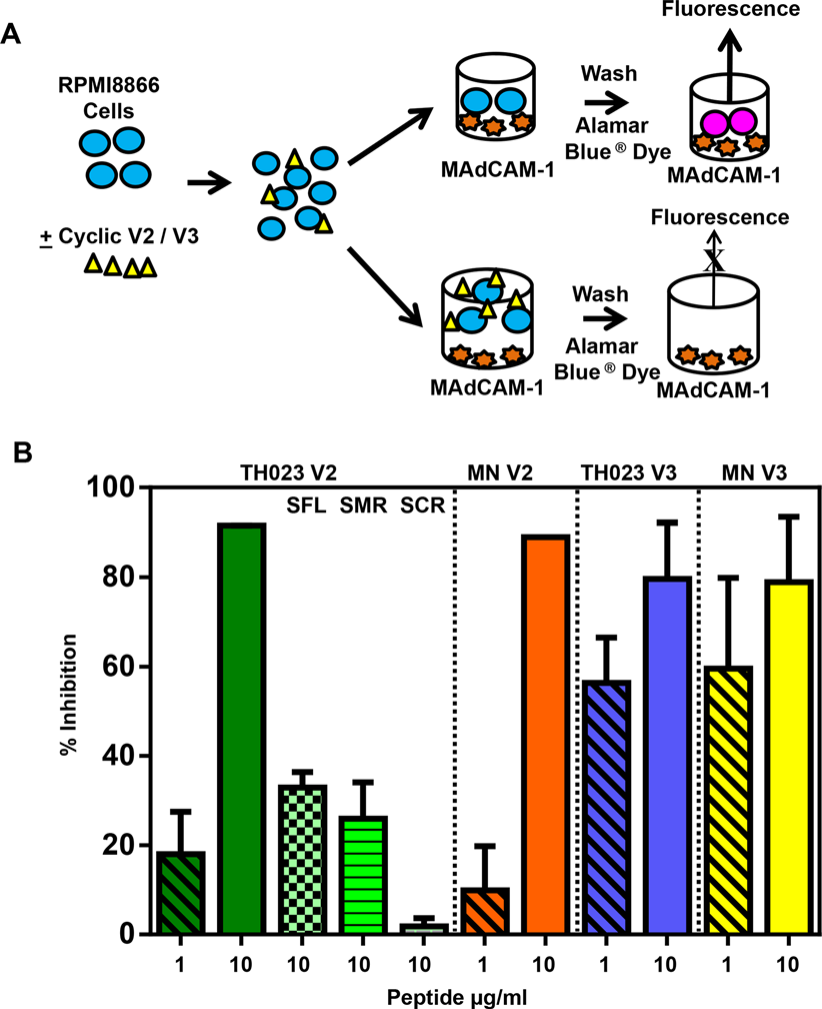
Competition of cyclic V2 and V3 peptides with MAdCAM-1 for binding to α4β7 integrin receptor. **(A)** Schematic of the assay set up. **(B)** Cyclic V2 and V3 peptides compete with MAdCAM-1 for binding to the integrin receptor. RPMI8866 cells were incubated with 1 (diagonal bars) or 10 μg/ml of cyclic V2 TH023 (solid green bar), cyclic V2 MN peptides (solid orange bar), cyclic V3 TH023 (solid blue bar) or cyclic V3 MN (solid yellow bar), respectively before being added to MAdCAM-1 coated plates. Data points represent the average fluorescence units ± SEM of 2–4 experiments done in triplicate. In separate experiments, RPMI8866 cells were also incubated with 10 μg/ml of cyclic V2 TH023 scrambled flank regions (SFL, green checkerboard bar), scrambled mid region (SMR, green horizontal bar) or fully scrambled (SCR, green brick bar) peptides respectively before being added to MAdCAM-1 coated plates. The binding of α4β7 on RPMI8866 cells to the fully scrambled peptide was lower than the binding to the scrambled mid region or the scrambled flank region. The data is shown as the average % inhibition ± SEM of 2 independent experiments done in triplicate.

The schematic for the peptide competition assay is shown in Fig 5A. RPMI8866 cells were pre-incubated with varying concentrations (1 or 10 μg/ml) of cyclic V2 and V3 (TH023 or MN) before being added to MAdCAM-1-coated plates (Fig 5B). A dose-dependent inhibition of α4β7 integrin binding to MAdCAM-1 was observed with all four of the peptides with approximately 80–90% inhibition at the 10 μg/ml concentration. In order to rule out the possibility of non-specific binding of proteins or sugars to RPMI8866 cells, HIV-1 p24, ovalbumin, or mannose, were added as controls in the competition assay (Table 2). Ovalbumin, p24, and mannose showed no significant inhibition.

In the RV144 immune correlates study, the vaccine-induced responses targeted multiple binding epitopes in the mid region of the V2 loop (aa 169–184) that contained the integrin-binding motif LDI/V, which is also the motif present in MAdCAM-1. The vaccine-induced responses were associated with a decreased proportion of infecting viruses carrying residue K169 and an increased proportion of infecting viruses carrying I181 [35]. Therefore, biotinylated cyclic V2 peptides with scrambled flanking regions (SFL), scrambled mid-region (SMR), or fully scrambled mid and flanking regions (SCR), were synthesized (Table 1) [6]. RMPI8866 cells were incubated with each of these peptides to map the V2 loop regions that bind to the integrin receptor and thus inhibit the binding to MAdCAM-1 (Fig 5B). In contrast to the 80–90% inhibition of binding of MAdCAM-1 to α4β7 on RPMI8866 cells observed with cyclic V2 and V3 TH023 peptides, 30–38% inhibition was obtained with SFL (green checkerboard bar) and SMR (green horizontal bar) V2 peptides (Fig 5B, first panel). The V2 SFL peptide retains the K169 and the I181 residues, whereas the V2 SMR peptide has K169Q and I181L. The fully scrambled V2 peptide, SCR showed less than 2% inhibition demonstrating that the V2 flanking and mid regions contribute to integrin receptor binding.

### Inhibition of binding of α4β7 to V2- and V3-peptides by specific mAbs

**Fig 6:**
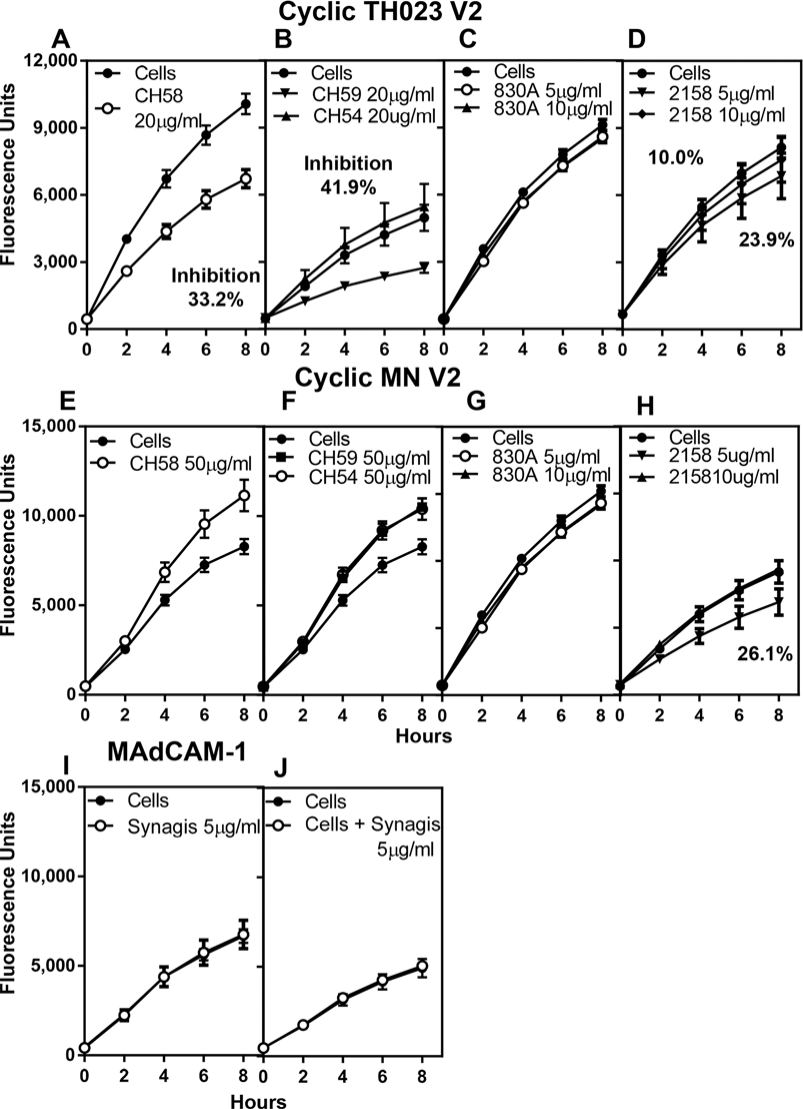
Inhibition of α4β7 integrin binding to cyclic V2 peptides by V2 mAbs. Human CH58, CH59, CH54, 830A, and 2158 mAbs were incubated with either cyclic V2 TH023 **(A-D)** or with cyclic V2 MN **(E-H)** peptides. CH58 **(A)**, CH59 **(B)** and 2158 **(D)** inhibit the binding of cyclic V2 TH023 peptide to α4β7 on RPMI8866 cells, while 830A **(C)** and a control mAb CH54 **(B)** did not inhibit the α4β7 binding. In contrast, only mAb 2158 **(H)** inhibited the binding of cyclic V2 MN to α4β7 on RPMI8866 cells. CH58 **(E)**, CH59 **(F)**, 830A **(H)** and CH54 **(F)** did not inhibit integrin binding to V2 MN. **(I and J)** Synagis^®^, a mAb specific to RSV was either incubated with the cells **(J)** and then added to MAdCAM-1 coated wells or **(I)** the mAb was added to MAdCAM-1 coated plates, followed by the addition of RPMI8866 cells. No inhibition of binding to α4β7 integrin was demonstrated in either case indicating the specificity of the assay. All experiments were performed 3 times. Each time point in graphs **A**, **B**, **E**, and **F**, represent the average fluorescence units ± S.D. of triplicate points of 3 experiments, while graphs **C**, **D**, **G**, **H**, **I**, and **J** are a representative plot of 3 experiments. The % inhibition if applicable is shown in the respective panels.

Since both cyclic V2 and V3 peptides bound α4β7 on RPMI8866 cells and inhibited the binding of α4β7 to its natural ligand MAdCAM-1, we next examined if human mAbs specific for V2 and V3 regions would prevent the binding of the peptides to α4β7 on RPMI8866 cells. Two V2-specific mAbs CH58 and CH59, which bind to the mid region of the V2 loop and are CRF01-AE specific, were used along with a control mAb (CH54), which does not bind to the V2 or V3 region. These 3 mAbs were isolated from RV144 vaccinees [33]. In addition, two V2 integrin binding (V2i) human mAbs, 830A and 2158 [36], which bind to gp120 and V1V2 fusion proteins [33], but not to linear V2 peptides were also tested [37]. All of these mAbs were incubated with cyclic V2 TH023 peptide-coated plates (Fig 6A, 6B, 6C and 6D) followed by the addition of RMPI8866 cells. The CH58 and CH59 mAbs inhibited the binding of RMPI8866 cells by 33% and 42%, respectively (Fig 6A and 6B). The control mAb CH54 (closed triangles) as expected did not inhibit the binding (Fig 6B). Of the two human V2i mAbs tested, 2158 inhibited the binding of cyclic V2 peptide to α4β7 on RPMI8866 cells by 24% (Fig 6D), while 830A did not inhibit the binding (Fig 6C). These 5 mAbs were also tested with cyclic V2 MN peptide (Fig 6E, 6F, 6G and 6H). CH58, CH59, and CH54 did not inhibit the binding of α4β7 on RPMI8866 cells to V2 MN peptide even at concentrations of 50 μg/ml (Fig 6E and 6F), thus demonstrating the specificity of inhibition obtained with the TH023 peptide, as the epitope that the mAbs CH58 and CH59 recognize are not present in the subtype B MN V2 cyclic peptide. Again, only mAb 2158 at a concentration of 5 μg/ml inhibited the binding of MN peptide to α4β7 on RPMI8866 cells by 26% (Fig 6H). As a control, Synagis^®^, a mAb against Respiratory Syncytial Virus (RSV) was added to MAdCAM-1 coated plates followed by the addition of RPMI8866 cells (Fig 6I) or RPMI8866 cells were preincubated with Synagis^®^ (Fig 6J) and then added to MAdCAM-1 coated plates. In both cases, Synagis^®^ did not inhibit the α4β7 binding thus demonstrating the specificity of the assay and that the mAb was not nonspecifically adhering to the surface of RPMI8866 cells.

**Fig 7:**
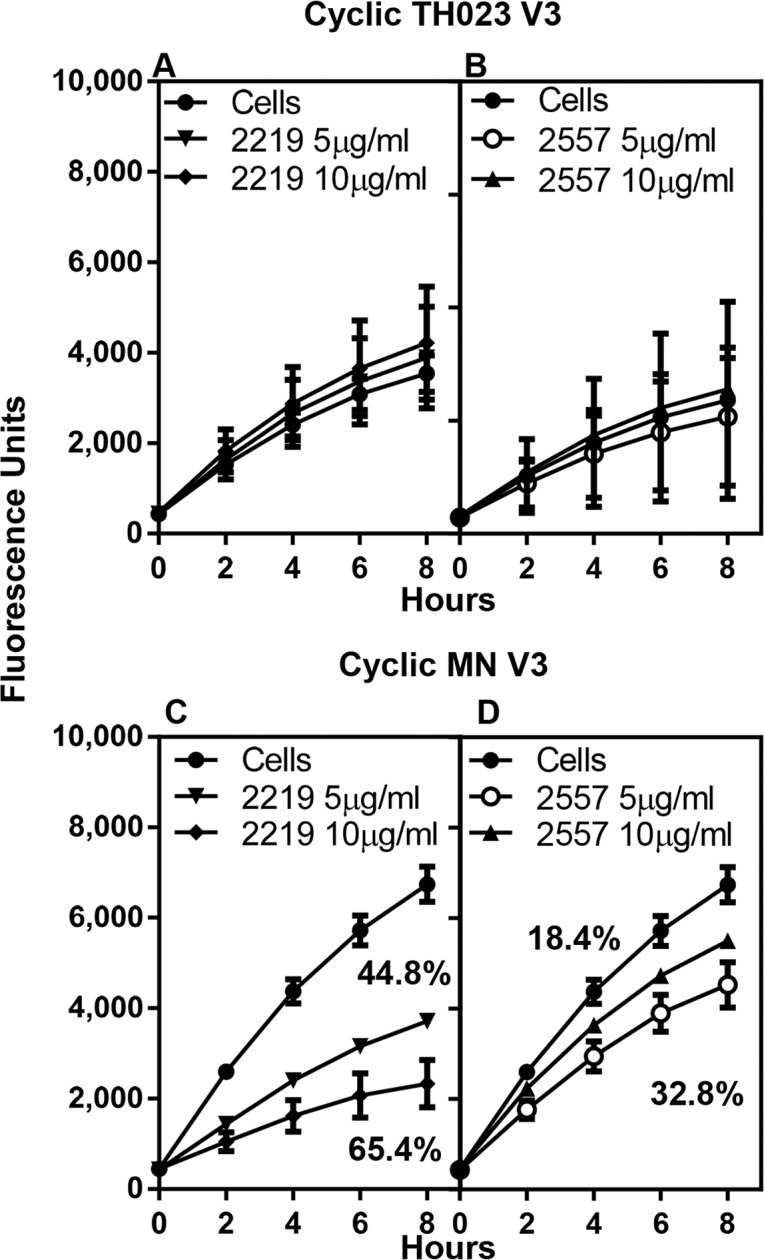
Inhibition of α4β7 integrin binding to cyclic V3 peptides by V3 mAbs. MAbs 2219 and 2557 were incubated with either cyclic V3 TH023 **(A and B)** or with cyclic V3 MN **(C and D)**. Neither mAbs 2219 **(A)** nor 2557 **(B)** inhibited the binding of cyclic V3 TH023 peptide to α4β7 on RPMI8866 cells. However, both mAb 2219 **(C)** and mAb 2557 **(D)** inhibited the binding of cyclic V3 MN to α4β7. Each time point represents the average fluorescence units of 2–3 experiments ± S.D. of triplicate points. The percent inhibition, if applicable, is shown in the respective panels.

Human V3-specific mAbs (2219 and 2557) were incubated with either cyclic V3 TH023 (Fig 7A and 7B) or V3 MN peptide-coated plates (Fig 7C and 7D). Although mAbs 2219 and 2557 did not inhibit the binding of α4β7 on RPMI8866 cells to cyclic V3 TH023 peptide, they did inhibit the binding to cyclic V3 MN peptide. MAb 2219 showed a dose-dependent inhibition of 45% and 65% at 5 μg/ml and 10 μg/ml of antibody respectively (Fig 7C), while mAb 2557 showed a slightly lower inhibition of 18% and 33% (Fig 7D). These results are in agreement with the results of Swetnam et al. [38] who showed that mAbs 2219 and 2557 bind CRF01_AE strains poorly, but recognize 70% of the circulating subtype B strains.

### Inhibition of α4β7 binding to cyclic V2 peptides by plasma from naturally infected (CRF01_AE) individuals and by IgG from RV144 vaccinees

**Fig 8:**
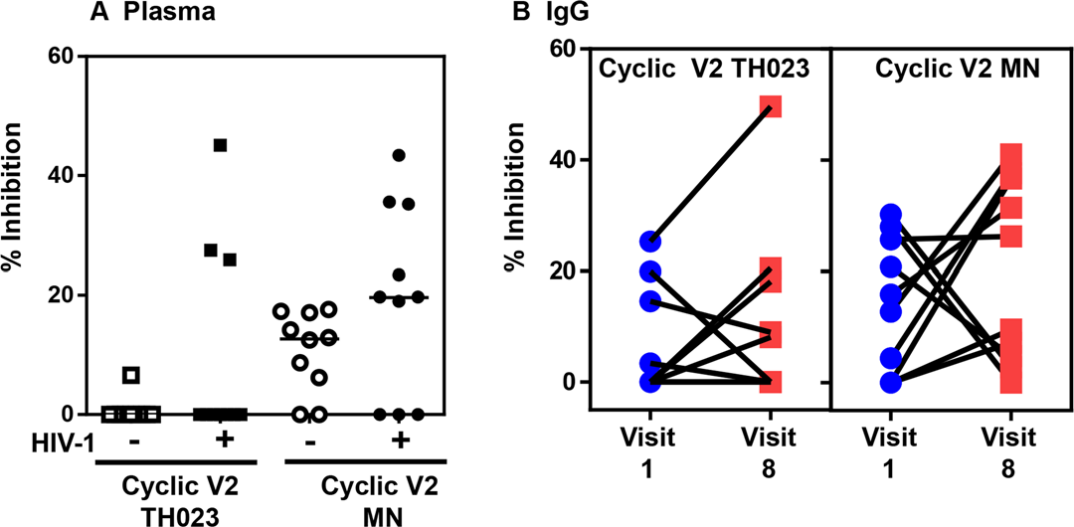
Plasma from uninfected, HIV-1-infected individuals, or purified IgG from uninfected RV144 vaccinees inhibit α4β7 integrin binding. Plasma (1:200 dilution) from Thai individuals uninfected or HIV-1 infected **(A)** or purified IgG from uninfected RV144 vaccinees **(B)** were incubated with cyclic V2 TH023 or MN peptides. **(A)** HIV-1 infected Thai individuals (3 out of 10) blocked the binding of α4β7 integrin to cyclic TH023 (closed squares). Plasma from uninfected (open circles) and infected individuals (closed circles) inhibited the binding of α4β7 integrin to cyclic MN V2 peptides to varying degrees; however the infected plasma exhibited higher levels of inhibition. **(B)** Purified IgG from uninfected RV144 vaccinees (post vaccination, Visit 8) inhibited the binding of α4β7 integrin to both TH023 and MN V2 peptides compared to pre-vaccination (Visit 1). The data shown are the mean of triplicate points.

Plasma samples from Thai blood donors uninfected or infected with CRF01_AE virus were studied to determine if plasma from uninfected or naturally infected individuals could inhibit α4β7 on RMPI8866 cells from binding to either cyclic V2-TH023 or V2-MN peptides. Plasma samples from 10 volunteers (diluted 1:200) were individually incubated with either cyclic V2-TH023 or V2-MN peptide-coated plates, washed, and followed by the addition of RPMI8866 cells (Fig 8A). The inhibition ranged from 0–45%. As expected, a higher degree of inhibition was obtained with plasma from infected compared to uninfected individuals.

Next we studied the ability of purified IgG from 10 uninfected RV144 vaccinees, pre (visit 1) and week 26 (2 weeks after last vaccination, visit 8), to inhibit α4β7 integrin binding to cyclic V2 TH023 and MN peptides (Fig 8B). Two different concentrations of purified IgG, 10 μg/ml and 20 μg/ml, were tested and the data from the 10 μg/ml is shown. Purified IgG inhibited cyclic TH023 and MN V2 peptide binding to α4β7 integrin receptor to varying levels in 4 and 6 out of the 10 vaccinees, respectively. Inhibition with purified IgG was also observed with V3 TH023 but not with V3 MN peptides (data not shown).

## Discussion

The α4β7 integrin receptor on the surface of T cells has been shown to interact with the V2 loop through the LDI/V motif [8]. CryoEM data suggest that the HIV-1 gp120 V1/V2 domain is located at the tip of the envelope spike, ~150 Å from the virus envelope thus favorably positioning it for receptor interactions. The α4β7 integrin receptor is about 3-times further extended than the primary CD4 receptor and hence readily accessible to capture the virus [39].

The RV144 immune correlates study has directed the focus of the HIV-1 field to the V1V2 and V3 regions of HIV-1 envelope protein gp120 [3–5]. However, the mechanism by which these antibodies exert their protection is currently unknown. It has been postulated that antibodies that bind to or near the integrin α4β7-binding motif probably interfere with the gp120-α4β7 interaction and prevent viral transmission [23, 40]. However, studies showing a lack of detectable α4β7 binding to HIV-1 virions and gp120 envelope proteins argue against this homing receptor being critical for mucosal HIV-1 transmission [41, 42]. In contrast, studies by Nakamura et al [43] demonstrated that antibodies to the V2 domain inhibited the binding of rgp120 to α4β7. Recently another region within the V2 loop (aa 170–172) has been identified that affects integrin binding to gp120 [44]. *In vivo* studies in rhesus macaques have demonstrated that targeting α4β7 by administering an anti-α4β7 monoclonal antibody during acute infection reduces mucosal transmission of SIV _mac251_ [20, 22].

Both in vitro and SIV transmission /infection studies have their own limitations. In the host mucosa, the virus has to be captured from a relatively small inoculum in a short period of time and the initial interactions between the viral envelope, CD4, and α4β7 might be crucial. The current *in vitro* assay that utilizes primary CD4^+^ T cells [45] for the detection of antibodies that bind to α4β7 must induce the active form of α4β7 with retinoic acid and block the CD4 receptor with anti-CD4 antibody in order to distinguish α4β7 binding from CD4 binding [8]. Theoretically, the number of α4β7 and CD4 receptors could vary depending upon the donor and the concentration of retinoic acid used. Studies utilizing a viral replication inhibition assay have suggested a role for α4β7 in HIV infection, which is influenced not only by the sequence of the α4β7 binding motif but also by other viral and host factors [46]. Several studies have used anti-α4 or anti-β7 antibody [41, 42, 47] to demonstrate the lack of interaction between α4β7 integrin receptor and gp120 or virions. However, these antibodies may not bind the activated form of α4β7 integrin receptor. To our knowledge, ACT-1 is the only mAb that is capable of binding all forms of the activated α4β7 integrin receptor.

In the RV144 Phase III trial, the majority of the antibody responses to the V2 region were directed against the mid-region of the V2 loop, which included the LDI/V motif [1]. Antibody responses to the cyclic V2 SFL peptide as measured by ELISA or Biacore were almost identical to those against the wild type cyclic V2 peptide, indicating that the majority of V2 antibody responses were localized within this region. Scrambling the amino acid sequences of the mid-region of the V2 loop (SMR) significantly reduced the magnitude and frequency of antibody binding with only 50% of the plasma samples being above background values [6]. However unlike the RV144 ELISA/Biacore binding data mentioned above, both the SFL and SMR V2 peptides specifically inhibited (35–40%) the binding of α4β7 to MAdCAM-1 as shown by the absence of inhibition by the completely scrambled V2 peptide (SCR). Thus, these data suggest that in addition to the mid region, amino acid sequences in the flanking region can also influence the V2-α4β7 interaction.

Surprisingly, both MN and TH023 V3 peptides strongly inhibited (80%) the binding of MAdCAM-1 to α4β7 receptors. The inhibition was specific as the interaction could be blocked by ACT-1. The MN and TH023 V3 peptide sequences vary considerably between each other and their respective V2 sequences, thus providing additional support that besides the LDI motif, other regions in V2 and V3 are important for the α4β7-V2/V3 interaction. The binding could be due to charged amino acids and/or discontinuous epitopes in the highly dynamic V2/V3 loop regions. The direct intramolecular interaction between V2 and V3 loops stabilized by sulfated tyrosines within the V2 loop is further supported by biological and structural studies [48, 49]. Furthermore, the conformational flexibility of the V2 region is demonstrated by the different structures adopted when it is complexed with mAbs. V2 exists as an α-helical structure or as a coil when it is bound by CH58 or CH59, while it is in a β strand structure when bound by the mAb PG9 [33, 50]. Not only does the V2 exhibit multiple conformations, but the α4β7 receptor also exhibits at least 3 different conformations, which are in a dynamic flux [51] further contributing to the complexity of the α4β7 receptor-gp120 interaction.

Inhibition of α_4_β_7_ binding has been demonstrated with a panel of V2-specific mouse mAbs whose epitopes overlap the integrin-binding site [43]. The RV144-derived V2-specific mAbs, CH58 and CH59 recognize a continuous linear region of V2, amino acids 167–181 and 168–173 respectively, are glycan independent, and bind both monomeric gp120 and V2 peptides [33]. In our study, both CH58 and CH59 inhibited the binding of TH023 but not MN V2 peptides to α4β7 receptors.

Recently Mayr et al [52] and Upadhya et al. [36], have described the epitope and function of a set of mAbs designated as variable loop V2 integrin (V2i)-specific antibodies. V2i epitopes require proper conformation of the V1V2 domain and map to the disordered V2 loop that connects the C and D strands, in particular, the area overlapping with the integrin α4β7-binding site [53]. We tested two of these V2i mAbs, 830A and 2158 [52, 53] to determine if they could inhibit α4β7-binding. Although, these V2i mAbs do not bind to linear V2 peptides, similar to others previously described [37], in our study a modest inhibition was observed with mAb 2158. This is probably due to mAb 2158 binding poorly to the cyclic V2 peptide.

Anti-V3 antibodies are present in the sera of virtually all individuals infected with HIV-1 [54, 55]. Several V3-specific human mAbs have been produced [56–58] and these mainly target the crown region of V3 [59, 60]. Two of the V3 human mAbs, 2219 and 2257 that recognize epitopes in the crown region, aa 304–318 (see Table 1), specifically inhibited (65% and 33%, respectively) the V3 MN binding but not the V3 TH023 binding to the α4β7 integrin receptor. The lysine (K) residue at position 305 in the V3 peptide appears to be important to form potential salt bridges with the mAbs. Lysine is replaced by tyrosine (T) in V3 TH023 peptide and this amino acid change could be the reason for the observed lack of inhibition of the V3 TH023 peptide binding to the α4β7 integrin receptor by the two mAbs tested in our study. This would also suggest that residues 304–318 of the V3 loop are involved in binding to the α4β7 integrin receptor.

Based on neutralization data, it is believed that V1V2 and V3 epitopes are not readily accessible on the envelope spikes [36, 61]. Recently published trimeric HIV-1 envelope structures [62–65] and previously published electron tomography and cryoelectron microscopy studies [63, 66–68] have revealed that V1V2 is located at the apex of the unliganded trimer and should theoretically be accessible to anti-V2 antibodies, while the V3 loop is tucked below the V1V2 with the crown region facing away from the trimer surface. However, it is important to know if the V2 and V3 regions are accessible on the surface of the virions to interact with α4β7 integrin receptor. Therefore, we assessed the ability of primary HIV-1 and full-length IMCs from acute and chronic HIV-1 to inhibit the binding of α4β7 receptors to MAdCAM-1. Tier 1 viruses in general have a more open conformation of the trimers, with the V2 and V3 regions being more accessible to binding ligands. BaL, a subtype B virus has been shown to bind to both activated CD4^+^ T cells as well as cell lines stably expressing α4β7. The binding as well as the infectivity of α4β7-activated CD4^+^ T cells was decreased in the presence of anti- α4β7 antibodies [69]. Of the 4 subtype B tier 1 primary viruses tested in our assay, two of the viruses, SF162 and TH023 inhibited the binding in a dose-dependent manner, while US-1 and JRFL did not. JRFL gp120 and JRFL pseudovirus have been previously shown not to bind α4β7 or inhibit infection in the presence of mAbs ACT-1 or α4 specific mAb 2B4 [40, 41], while SF162 has been shown to inhibit the binding and infection [41] of the trimers, with the V2 and V3 regions being more accessible to binding ligands.

The two primary subtype B viruses, SF162 and TH023, that were able to inhibit α4β7-binding in our study are predicted to have a coiled structure and an α-helix in the 166–169 region of V2 while JRFL and US-1 that did not inhibit do not have a predicted coiled structure or α-helix. JRFL and US-1 are predicted to have a broken α-helix structure and a β-turn structure, respectively. This specific amino acid region of V2 contains both the sieve mutation at position 169 [35] and a cryptic determinant of α4β7 integrin binding (aa166-178). Coincidentally, all of the IMC’s tested in our system are predicted to have α-helixes at residures166-169. Furthermore, in our assay, acute and chronic CRF01_AE, and C showed a trend of higher inhibition with increasing concentrations of the virus. In all likelihood, the predicted structures alone are not then the only measure of α4β7 integrin inhibition, but rather a range of factors including amino acid charge that could also contribute to the binding/inhibition.

During the course of HIV-1 infection, binding antibodies to various regions of the envelope protein are induced. Our studies indicate that plasma from chronic HIV-1 infected individuals contain antibodies directed against the V2 region that inhibit the binding of α4β7 to the V2 peptides to varying degrees. Interestingly, purified IgG from RV144 vaccinees also inhibited the binding of α4β7 to V2 TH023 and MN peptides, demonstrating that vaccination can induce these types of antibodies.

## Conclusion

Besides the known LDI motif, our study demonstrates previously unidentified regions in HIV-1 V2 and V3 loops that are important in α4β7 binding. As has been previously suggested, a combination of structure and charge within V2 may be the critical features modulating the effect of the α4β7-gp120 interaction. V2- and V3-specific binding mAbs were able to block the α4β7 binding. Epitopes involved in binding to the α4β7 region were accessible on PBMC produced primary infectious viruses and on acute infectious molecular clone viruses. From these data, we hypothesize that early induction of V2 specific antibodies targeting the α4β7 region as a result of vaccination can play a role in viral acquisition. Understanding the interaction of V2 and V3 regions of HIV-1 envelope with α4β7 will contribute to the selection and design of immunogens that can induce antibodies to these regions with protective functions.

## Supporting Information

S1 Data Flow Cytometry Raw Data for Fig 1A APC-α4β7. (FCS)

S2 Data Flow Cytometry Raw Data for Fig 1A Isotype Control. (FCS)

S3 Data Flow Cytometry Raw Data for Fig 1A Unstained. (FCS)

S4 Data Flow Cytometry Raw Data for Fig 1B Goat-anti-mouse IgG Texas Red. (FCS)

S5 Data Flow Cytometry Raw Data for Fig 1B gp120 Protein. (FCS)

S6 Data Flow Cytometry Raw Data for Fig 1C Scrambled V2 Peptide 1μg. (FCS)

S7 Data Flow Cytometry Raw Data for Fig 1C 92TH023 V2 Peptide 1μg. (FCS)

S8 Data Flow Cytometry Raw Data for Fig 1C Unstained Cells. (FCS)

S9 Data Flow Cytometry Raw Data for Fig 1C Scrambled V2 Peptide 5μg. (FCS)

S10 Data Flow Cytometry Raw Data for Fig 1C 92TH023 V2 Peptide 5μg. (FCS)
